# Metagenome-assembled genome (MAG) of *Oceancaulis alexandrii* NP7 isolated from Mediterranean Sea polluted marine sediments and its bioremediation potential

**DOI:** 10.1093/g3journal/jkab210

**Published:** 2021-06-26

**Authors:** Filippo Dell’Anno, Leonardo Joaquim van Zyl, Marla Trindade, Christophe Brunet, Antonio Dell’Anno, Adrianna Ianora, Clementina Sansone

**Affiliations:** 1 Stazione Zoologica Anton Dohrn, Istituto Nationale di Biologia, Ecologia e Biotecnologie marine, 80121 Naples, Italy; 2 Department of Biotechnology, Institute for Microbial Biotechnology and Metagenomics (IMBM), University of the Western Cape, 7535 Bellville, Cape Town, South Africa; 3 Department of Life and Environmental Science, Università Politecnica delle Marche, 6031 Ancona, Italy

**Keywords:** *Oceanicaulis alexandrii*, metagenome assembled genome, bioremediation, Mediterannean Sea

## Abstract

*Oceanicaulis alexandrii* strain NP7 is a marine bacterium which belongs to the *Hyphomonadaceae* family and was isolated from sediments highly contaminated with metals and polycyclic aromatic hydrocarbons released for decades by industrial activities in the Gulf of Naples (Mediterranean Sea). Here, we report the partial genome sequence and annotation of *O. alexandrii* strain NP7 that contains a chromosome of 2,954,327 bp and encodes for 2914 predicted coding sequences (CDSs) and 44 RNA-encoding genes. Although the presence of some CDSs for genes involved in hydrocarbon degradation processes (*e.g., alkB*) have already been described in the literature associated with the *Oceanicaulis*, this is the first time that more than 100 genes involved in metal detoxification processes and hydrocarbon degradation are reported belonging to this genus. The presence of a heterogeneous set of genes involved in stress response, hydrocarbon degradation, heavy metal resistance, and detoxification suggests a possible role for *O. alexandrii* NP7 in the bioremediation of these highly contaminated marine sediments.

## Introduction

Coastal sediments subjected to high anthropogenic pressure can accumulate large amounts of chemical contaminants (Tamburini *et al.* 2020) which can have detrimental consequences on ecosystem and human health (Ali *et al.* 2019; Rasheed *et al.* 2019). Thus, there is an urgent need to find sustainable and eco-compatible solutions able to efficiently recover and remediate contaminated marine sediments. Among these, eco-friendly strategies based on bioremediation are gaining increasing attention for their potential in the clean-up of contaminated marine coastal ecosystems (Catania *et al.* 2015; Brar *et al.* 2017).

Several microbes involved in the removal of pollutants have been identified and include bacteria belonging to the genera *Alcaligens*, *Bacillus*, *Enterobacter*, *Flavobacterium*, *Pseudomonas* (Ojuederie and Babalola 2017; Wahhab *et al.* 2021), *Achromobacter*, *Acinetobacter*, *Alteromonas*, *Arthrobacter*, *Burkholderia* (Xu *et al.* 2018), and obligate hydrocarbonoclastic bacteria such as *Alcanivorax*, *Thallassolituus*, *Cycloclasticus*, *Oleispira* (Yakimov *et al.* 2007). The development of high throughput sequencing technologies has provided new insights and information on new microorganisms with potential for the bioremediation of contaminated marine matrices (Czaplicki and Gunsch 2016).

Here, we describe the metagenome-assembled genome (MAG) of *Oceanicaulis alexandrii* strain NP7 isolated from highly contaminated sediments of the Bagnoli-Coroglio bay in the Gulf of Naples (Southern Tyrrhenian Sea, Mediterannean Sea).

This site is a typical example of a coastal area chronically contaminated by different pollutants (*i.e.*, metals, aliphatic and aromatic hydrocarbons and polychlorinated biphenyl) which have been released for decades by industrial activities and stopped at the beginning of 90s (Romano *et al.* 2009, 2018). As annotation of the *O.* *alexandrii* strain NP7 genome has highlighted the presence of numerous genes involved in hydrocarbon degradation and heavy metal detoxification, results are discussed in the framework of its bioremediation potential.

## Materials and methods

### Sample collection and bacteria isolation

The superficial sediment samples (0–20 cm) used for the isolation of bacteria were collected in November 2017 with a Van Veen grab in the Bagnoli-Coroglio area (Gulf of Naples): 40°48′29.0″N 14°09′54.2″E. Duplicate samples were immediately placed into sterile bags (Whirl-Pak, Nasco) and stored at 4 °C in the dark, until their processing in the laboratory.

The sediment was plated on Marine Agar (MA) (Bacto-Agar, Difco) and incubated at 28°C for 48 h.

### DNA extraction, Illumina sequencing, and gene annotation

For genome sequencing, DNA was extracted from bacterial consortia, following sequential dilution on agar plates, with the DNeasy Blood & Tissue kit (Qiagen), according to the manufacturer’s instructions. DNA concentration was determined using a Qubit fluorometer (Thermo-Fisher). Sequence library preparation was performed using the Nextera DNA Flex kit (Illumina, Hayward, USA) with 1 ng input DNA according to the manufacturer’s instructions. The resulting libraries were sequenced on the Illumina MiSeq platform at the University of Western Cape (South Africa) sequencing facility using a MiSeq Reagent kit V2 (500 cycle) with a 10% phiX v3 spike generating 2 × 250 bp reads per sample. Metagenome assembly was performed using CLC Genomics Workbench version 6.5. The raw reads were trimmed and demultiplexed, and ≤500 bp contigs were removed from the final assembly. Binning of metagenomic contigs was performed using MyCC (Lin and Liao 2016), while completeness and contamination of MAG as well as genome quality were determined using CheckM using the lineage-specific workflow and default parameters (Parks *et al.* 2015). The CGView (Circular Genome Viewer) software was used to obtain a circularized map of the chromosome, using a default server setting (Stothard and Wishart 2005). Gene prediction and annotation were performed by Rapid Annotation using the Subsystem Technology (RAST) (http://rast.nmpdr.org/) (Overbeek *et al.* 2014) and MicroScope pipelines (Vallenet *et al.* 2017).

### Taxonomic analysis

The Genome Taxonomy Database (https://gtdb.ecogenomic.org/) implemented through K-Base (www.kbase.us) was used to perform the classification.

### Data availability

The complete genome sequence of *O.* *alexandrii* NP7 has been deposited in the GenBank database under number JADWML000000000.1 and bioproject number PRJNA669418. Sequence read archives are available at SRR13577792.

Supplementary material is available at *G3* online.

## Results and discussion

Metagenome sequencing of the bacterial consortium cultured on marine agar, followed by binning of the metagenome contigs highlighted the dominance of MAGs representing three different bacterial species: *Halomonas* sp. SZN1, *Epibacterium* sp. SZN4, and *O.* *alexandrii* NP7. Since the phylogenetic classification and the ability to degrade hydrocarbons and reduce the toxicity of metals for *Halomonas* sp. SZN1, and *Epibacterium* sp. SZN4 has already been described in Dell’ Anno *et al.* (2020), the focus of this work was on the description of the *O.* *alexandrii* NP7 MAG (Table 1) and its bioremediation potential.

**Table 1:** *Oceanicaulis alexandrii NP7*: general features and MIGS mandatory information

Item	Description
Classification	Domain bacteria
	Phylum Proteobacteria
	Class *Alphaproteobacteria*
	Order *Caulobacterales*
	Family *Maricaulaceae*
	Genus *Oceanicaulis*
	Species *Oceanicaulis alexandrii*
Investigation type	Bacteria
Project name	*Oceanicaulis alexandrii* strain: NP7
Collection date	May 10, 2018
Geographic location (latitude and location)	40°48′28.6″N 14°10′03.0″E
Geographic location (country and/or sea, region)	Gulf of Naples, Mediterranean Sea, Italy
Sediment environmental package	Sediment
Environment (biome)	Sea
Environment (feature)	Sediments
Environment (material)	Sea sediments
Depth	−4 m
Elevation	0 m
Number of replicons	1
Relationship to oxygen	Aerobic
Sample collection device or method	Van veen grab
Sequencing method	Illumina MiSeq
Assembly	CLC NGS Cell 6.5

Furthermore, we have chosen to thoroughly analyze the genome of *O.* *alexandrii* NP7 as little information is available in the literature on the biotechnological potential of members belonging to the *Hyphomonadaceae* family, and to our knowledge, no member of *O.* *alexandrii* has ever been used for bioremediation purposes.

**Figure 1:**
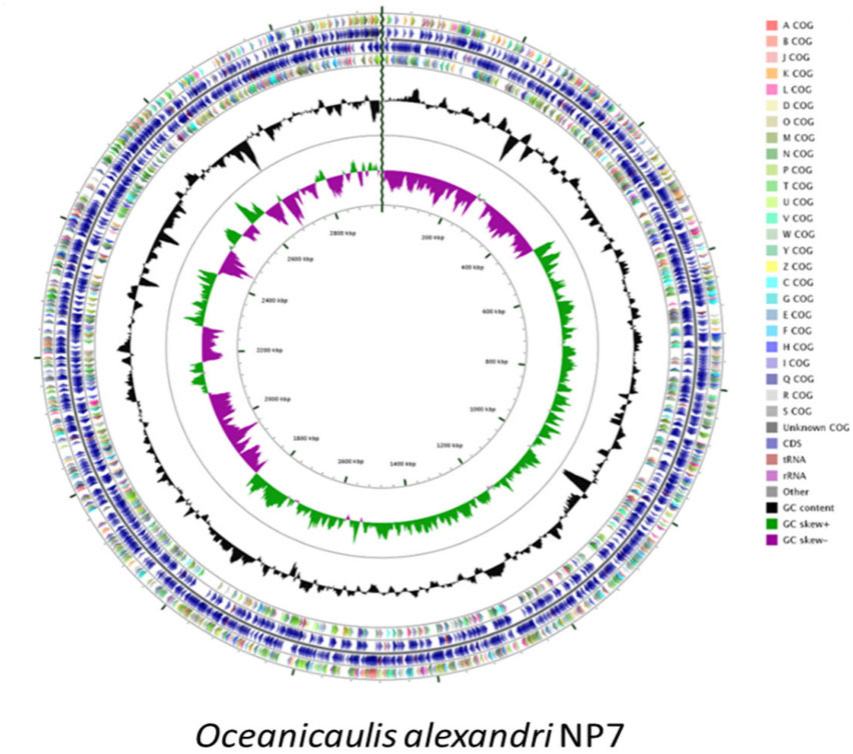
Circular representation of the *Oceanicaulis alexandrii* NP7 genome. The different rings represent (from outer to inner) predicted protein-coding sequences (CDS) on the forward (outer wheel) and the reverse (inner wheel) strands (circle 2 and 3) colored according to the assigned COG classes (circle 1, 4), G+C content (circle 5), GC skew (circle 6), genomic position (circle 7). The COG colors represent functional groups (A, RNA processing and modification; B, chromatin structure and dynamics; J, Translation, ribosomal structure and biogenesis; K, Transcription; L, Replication, recombination and repair; D, Cell cycle control, cell division, chromosome partitioning; O, Posttranslational modification, protein turnover, chaperones; M, Cell wall/membrane/envelope biogenesis; N, Cell motility; P, Inorganic ion transport and metabolism; T, Signal transduction mechanisms; U, Intracellular trafficking, secretion, and vesicular transport; V, Defense mechanisms; W, Extracellular structures; Y, Nuclear structure; Z, Cytoskeleton; C, Energy production and conversion; G, Carbohydrate transport and metabolism; E, Amino acid transport and metabolism; F, Nucleotide transport and metabolism; H, Coenzyme transport and metabolism; I, Lipid transport and metabolism; Q, Secondary metabolites biosynthesis, transport and catabolism; R, General function prediction only; S, Function unknown).

The draft genome of *O.* *alexandrii* NP7, an Alphaproteoacteria belonging to the Order Caulobacterales, contains 2,954,327 bases (Figure 1).

The closest reference strain on the genomic taxa database, based on an Average Nucleotide Identity score of 97.5% is *O.* *alexandrii* DSM 11625, and on this basis, we have assigned the name *O.* *alexandrii* NP7. CheckM analysis showed a completeness of 96.5% (27 markers missing) and a contamination of 0.32% (1 marker duplicated) (Table 2).

**Table 2:** Genome statistics of *Oceanicaulis alexandrii* NP7

Genome features	%
CheckM completeness	96.5%
CheckM Contamination	0.3%
Size, bp	2,954,327
G+C content, %	62.71%
N50	209292
L50	2
Number of contigs	40
Number of subsystems	383
Number of coding sequences	2837
Function assigned	1995
Hypothetical	842
Number of RNAs	44

The draft genome sequence for *O.* *alexandrii* NP7 has a GC content of 62.71%, and is composed of 40 contigs and 2914 predicted coding sequences (CDSs) with an average length of 937.58 bp having a protein coding density of 91.51%. Of the total predicted CDSs, 1995 (69.4%) were assigned to a function, 842 (29.3%) were classified as hypothetical and 44 (1.5%) as coding for RNAs (Table 2). Seven Genomic Islands (GIs) were predicted using the integration of IslandPath-DIMOB, SIGI-HMM, and Island Pick provided IslandViewer4 (Bertelli *et al.* 2017), with a total of 129,287 bp (4.39% of the genome) and 151 predicted CDSs of which 42 were classified as proteins of unknown function (Supplementary Table S1). Predicted CDSs encode for regulators, transmembrane proteins, multi-drug resistance genes, exopolysaccharide production proteins, and oxidoreductases. Among these GIs, 12 mobile genetic elements, such as integrase/recombinase, and transposase genes were found, suggesting that these GIs can self-mobilize and promote horizontal gene transfer. To further explore the ability of *O.* *alexandrii* NP7 to survive in highly contaminated environments, we annotated and analyzed the gene functional categories (Supplementary Table S2). Thirty-seven genes related to “Virulence, Disease and Defense” functions, thus likely involved in playing a role in the resistance to antibiotics and toxic compounds. Thirty-one genes belonged to the subcategory of resistance to antibiotics and toxic compounds. The RAST annotation system highlighted the presence of five genes involved in antibiotic resistance such as Beta-lactamase (three genes) and resistance to floroquinolones (two genes). A prevalance of genes involved in heavy metal detoxification were identified, such as copper homeostasis and tolerance (eight genes); cobalt-, zinc-, and cadmium- resistance (seven genes); mercuric reductase (one gene); a mercuric resistance protein (one gene) and multidrug resistance efflux pumps (six genes). The presence of genes for heavy metal resistance suggests that *O.* *alexandrii* NP7 may have evolved to cope with highly metal contaminated environments. This observation is corroborated by the presence of 33 genes coding for superoxide dismutase and glutathione-related pathways, known for their antioxidant and detoxification capacity (Espinosa-Diez *et al.* 2015). Since the production of exopolysaccharides favors the immobilization of metals through the formation of metal-exopolysaccharide complexes (Gupta and Diwan 2017), the presence of 10 genes identified in the genome of *O.* *alexandrii* NP7—5 coding for dTDP rhamnose synthesis and 5 coding for rhamnose containing glycans—suggests that this species may have a potential role in the bioremediation of a heavy metal-contaminated matrix.

The annotations generated by the RAST and MicroScope pipelines revealed the presence of numerous genes involved in the degradation of hydrocarbons and polycyclic aromatic compounds, *e.g.*, genes involved in the degradation of the following hydrocarbons: cholorocyclohexane (two genes), benzoate (seven genes), fluorobenzoate (one gene), toluene (two genes), chloroalkane and chloroalkene (two genes), naphtalene (two genes), aminobenzoate (four genes), and ethylbenzene (one gene). Furthermore, 14 genes coding for pathways involved in the metabolism of central aromatic intermediates such as the catechol branch of the beta ketoadipate pathway and salycilate, gentisate, and homogentisate pathways, were identified.

The possibility of using *O.* *alexandrii* in bioremediation processes has previously been suggested by Oh *et al.* (2011), on the basis of the identification of the epoxide hydrolase, cyclohexanone monooxygenase, 6-hexanolactone hydrolase, and phytase genes. Here, we expand the identification to include 100 genes in the *O.* *alexandrii* NP7 genome proposed to be involved in the degradation of hydrocarbons, and in mechanisms involved in the detoxification of metals. This further emphasizes the potential ability of this bacterial strain to efficiently degrade organic contaminants and/or to be highly tolerant/resistant to metal contamination, and thus useful for bioremediation purposes.

## Author contributions

C.S. and F.D. carried out the *in situ* samplings and the laboratory experiments. F.D., L.J.v.Z., and M.T. performed the molecular/bioinformatics analysis and analyzed the results. F.D., C.B., L.J.v.Z., M.T., A.I., C.S., and A.D., interpreted the results. F.D. drafted the manuscript, all authors revised and approved the final manuscript.

## Funding

F.D. was funded by a PhD grant from the SZN and UNIVPM. This study was supported by the projects ABBaCo funded by the Italian Ministry for Education, University and Research (grant number C62F16000170001), Ocean Medicines (H2020-MSCA-RISE-2015), and MERCES (H2020-SC5-2015, grant number 689518).

## Conflict of interest

The authors declare that the research was conducted in the absence of any commercial or financial relationships that could be construed as a potential conflict of interest.

## Supplementary Material

jkab210_Supplementary_Data
