# Supplementary material for: Metagenome-assembled genome (MAG) of Oceancaulis alexandrii NP7 isolated from Mediterranean Sea polluted marine sediments and its bioremediation potential
Source: G3 (Bethesda). 2021 Jun 26;11(9):jkab210. doi: 10.1093/g3journal/jkab210 (PMC8496225; doi:10.1093/g3journal/jkab210)
Supplement: jkab210_Supplementary_Data [file jkab210_supplementary_data.docx]

***Supplementary Information***

**Supplementary Table 1**: List of the identified GIs and related gene function.

| **Island no.** | **Island start** | **Island end** | **Gene start** | **Gene end** | **Strand** | **Product** |
| --- | --- | --- | --- | --- | --- | --- |
| 1 | 262163 | 300415 | 262163 | 262909 | -1 | Serine/threonine protein phosphatase 1 |
|  | 262163 | 300415 | 262943 | 265174 | -1 | Capsular biosynthesis protein |
|  | 262163 | 300415 | 265259 | 266533 | -1 | Outer membrane protein/protective antigen OMA87 |
|  | 262163 | 300415 | 266632 | 267099 | -1 | putative PE-PGRS family protein PE_PGRS8 |
|  | 262163 | 300415 | 267151 | 267249 | -1 | protein of unknown function |
|  | 262163 | 300415 | 267284 | 269110 | -1 | conserved membrane protein of unknown function |
|  | 262163 | 300415 | 269132 | 269722 | -1 | Polysaccharide biosynthesis protein |
|  | 262163 | 300415 | 269658 | 269780 | 1 | protein of unknown function |
|  | 262163 | 300415 | 270120 | 270704 | 1 | conserved protein of unknown function |
|  | 262163 | 300415 | 270658 | 270846 | 1 | protein of unknown function |
|  | 262163 | 300415 | 270846 | 271121 | 1 | conserved protein of unknown function |
|  | 262163 | 300415 | 271393 | 272340 | 1 | conserved protein of unknown function |
|  | 262163 | 300415 | 272465 | 273772 | 1 | UDP-glucose 6-dehydrogenase |
|  | 262163 | 300415 | 273769 | 274737 | 1 | Uncharacterized 37.6 kDa protein in cld 5'region |
|  | 262163 | 300415 | 274816 | 275529 | 1 | SAM-dependent methyltransferase |
|  | 262163 | 300415 | 275548 | 276708 | -1 | conserved protein of unknown function |
|  | 262163 | 300415 | 276708 | 277379 | -1 | conserved protein of unknown function |
|  | 262163 | 300415 | 277376 | 278224 | -1 | conserved protein of unknown function |
|  | 262163 | 300415 | 278221 | 279417 | -1 | conserved protein of unknown function |
|  | 262163 | 300415 | 279837 | 281060 | 1 | conserved protein of unknown function |
|  | 262163 | 300415 | 281057 | 282361 | 1 | conserved protein of unknown function |
|  | 262163 | 300415 | 282378 | 283049 | 1 | conserved protein of unknown function |
|  | 262163 | 300415 | 283185 | 284423 | 1 | conserved protein of unknown function |
|  | 262163 | 300415 | 284416 | 285666 | 1 | putative Glycosyltransferase involved in cell wall bisynthesis |
|  | 262163 | 300415 | 285663 | 287216 | 1 | membrane protein of unknown function |
|  | 262163 | 300415 | 287224 | 288357 | -1 | conserved protein of unknown function |
|  | 262163 | 300415 | 288477 | 289892 | 1 | conserved membrane protein of unknown function |
|  | 262163 | 300415 | 289861 | 290871 | -1 | Acyltransferase 3 |
|  | 262163 | 300415 | 290854 | 290979 | 1 | protein of unknown function |
|  | 262163 | 300415 | 291083 | 291769 | -1 | Exopolysaccharide production protein ExoY |
|  | 262163 | 300415 | 291962 | 292138 | -1 | protein of unknown function |
|  | 262163 | 300415 | 292141 | 292329 | 1 | protein of unknown function |
|  | 262163 | 300415 | 293013 | 294344 | 1 | Methyltransferase |
|  | 262163 | 300415 | 294547 | 294855 | 1 | protein of unknown function |
|  | 262163 | 300415 | 294848 | 296263 | 1 | conserved protein of unknown function |
|  | 262163 | 300415 | 296769 | 297032 | 1 | protein of unknown function |
|  | 262163 | 300415 | 297043 | 297456 | 1 | conserved protein of unknown function |
|  | 262163 | 300415 | 297154 | 297321 | -1 | protein of unknown function |
|  | 262163 | 300415 | 297453 | 299090 | 1 | Recombinase |
|  | 262163 | 300415 | 299732 | 300415 | 1 | conserved protein of unknown function |
| 2 | 281057 | 285666 | 279837 | 281060 | 1 | conserved protein of unknown function |
|  | 281057 | 285666 | 281057 | 282361 | 1 | conserved protein of unknown function |
|  | 281057 | 285666 | 282378 | 283049 | 1 | conserved protein of unknown function |
|  | 281057 | 285666 | 283185 | 284423 | 1 | conserved protein of unknown function |
|  | 281057 | 285666 | 284416 | 285666 | 1 | putative Glycosyltransferase involved in cell wall bisynthesis |
|  | 281057 | 285666 | 285663 | 287216 | 1 | membrane protein of unknown function |
| 3 | 290854 | 297321 | 289861 | 290871 | -1 | Acyltransferase 3 |
|  | 290854 | 297321 | 290854 | 290979 | 1 | protein of unknown function |
|  | 290854 | 297321 | 291083 | 291769 | -1 | Exopolysaccharide production protein ExoY |
|  | 290854 | 297321 | 291962 | 292138 | -1 | protein of unknown function |
|  | 290854 | 297321 | 292141 | 292329 | 1 | protein of unknown function |
|  | 290854 | 297321 | 293013 | 294344 | 1 | Methyltransferase |
|  | 290854 | 297321 | 294547 | 294855 | 1 | protein of unknown function |
|  | 290854 | 297321 | 294848 | 296263 | 1 | conserved protein of unknown function |
|  | 290854 | 297321 | 296769 | 297032 | 1 | protein of unknown function |
|  | 290854 | 297321 | 297043 | 297456 | 1 | conserved protein of unknown function |
|  | 290854 | 297321 | 297154 | 297321 | -1 | protein of unknown function |
| 4 | 1017917 | 1037945 | 1017917 | 1018393 | -1 | Acetyltransferase (GNAT) family protein |
|  | 1017917 | 1037945 | 1018548 | 1018874 | -1 | conserved protein of unknown function |
|  | 1017917 | 1037945 | 1018957 | 1019409 | -1 | conserved protein of unknown function |
|  | 1017917 | 1037945 | 1019671 | 1020606 | 1 | protein of unknown function |
|  | 1017917 | 1037945 | 1020696 | 1021499 | -1 | conserved protein of unknown function |
|  | 1017917 | 1037945 | 1021569 | 1022363 | -1 | conserved protein of unknown function |
|  | 1017917 | 1037945 | 1022356 | 1025385 | -1 | Type I restriction enzyme R Protein |
|  | 1017917 | 1037945 | 1025385 | 1026542 | -1 | conserved protein of unknown function |
|  | 1017917 | 1037945 | 1026532 | 1028019 | -1 | Type I restriction enzyme M protein |
|  | 1017917 | 1037945 | 1028183 | 1028728 | 1 | conserved protein of unknown function |
|  | 1017917 | 1037945 | 1029062 | 1029700 | 1 | Integrase/recombinase XerD |
|  | 1017917 | 1037945 | 1029717 | 1030262 | -1 | protein of unknown function |
|  | 1017917 | 1037945 | 1030343 | 1030705 | -1 | protein of unknown function |
|  | 1017917 | 1037945 | 1030807 | 1031229 | -1 | protein of unknown function |
|  | 1017917 | 1037945 | 1031449 | 1032723 | -1 | Phage integrase family protein |
|  | 1017917 | 1037945 | 1032720 | 1033223 | -1 | conserved protein of unknown function |
|  | 1017917 | 1037945 | 1033399 | 1036179 | -1 | preprotein translocase subunit, ATPase |
|  | 1017917 | 1037945 | 1036297 | 1037289 | 1 | Peptidylprolyl isomerase |
|  | 1017917 | 1037945 | 1037350 | 1037775 | 1 | CTP pyrophosphohydrolase |
|  | 1017917 | 1037945 | 1037814 | 1037945 | 1 | Flp family type IVb pilin (fragment) |
| 5 | 1018548 | 1030705 | 1018548 | 1018874 | -1 | conserved protein of unknown function |
|  | 1018548 | 1030705 | 1018957 | 1019409 | -1 | conserved protein of unknown function |
|  | 1018548 | 1030705 | 1019671 | 1020606 | 1 | protein of unknown function |
|  | 1018548 | 1030705 | 1020696 | 1021499 | -1 | conserved protein of unknown function |
|  | 1018548 | 1030705 | 1021569 | 1022363 | -1 | conserved protein of unknown function |
|  | 1018548 | 1030705 | 1022356 | 1025385 | -1 | Type I restriction enzyme R Protein |
|  | 1018548 | 1030705 | 1025385 | 1026542 | -1 | conserved protein of unknown function |
|  | 1018548 | 1030705 | 1026532 | 1028019 | -1 | Type I restriction enzyme M protein |
|  | 1018548 | 1030705 | 1028183 | 1028728 | 1 | conserved protein of unknown function |
|  | 1018548 | 1030705 | 1029062 | 1029700 | 1 | Integrase/recombinase XerD |
|  | 1018548 | 1030705 | 1029717 | 1030262 | -1 | protein of unknown function |
|  | 1018548 | 1030705 | 1030343 | 1030705 | -1 | protein of unknown function |
| 6 | 1142309 | 1153869 | 1142309 | 1143601 | 1 | Cytochrome b |
|  | 1142309 | 1153869 | 1143614 | 1144420 | 1 | Ubiquinol-cytochrome c reductase cytochrome c1 subunit |
|  | 1142309 | 1153869 | 1144513 | 1145121 | 1 | conserved protein of unknown function |
|  | 1142309 | 1153869 | 1145118 | 1145996 | 1 | S-methyl-5'-thioadenosine phosphorylase |
|  | 1142309 | 1153869 | 1146032 | 1146832 | 1 | conserved exported protein of unknown function |
|  | 1142309 | 1153869 | 1146972 | 1147391 | 1 | 5'-methylthioadenosine phosphorylase |
|  | 1142309 | 1153869 | 1147394 | 1147621 | 1 | Uncharacterized HTH-type transcriptional regulator AF_1793 |
|  | 1142309 | 1153869 | 1147673 | 1148305 | -1 | Phage repressor protein C with HTH and peptisase S24 domain |
|  | 1142309 | 1153869 | 1148372 | 1148788 | 1 | conserved protein of unknown function |
|  | 1142309 | 1153869 | 1148785 | 1149828 | 1 | Dihydroorotate dehydrogenase (quinone) |
|  | 1142309 | 1153869 | 1149825 | 1150406 | -1 | MATE family multidrug resistance protein (fragment) |
|  | 1142309 | 1153869 | 1150462 | 1151121 | -1 | MATE family multidrug resistance protein (fragment) |
|  | 1142309 | 1153869 | 1151165 | 1152160 | -1 | conserved protein of unknown function |
|  | 1142309 | 1153869 | 1152200 | 1152622 | 1 | putative DCC family thiol-disulfide oxidoreductase YuxK |
|  | 1142309 | 1153869 | 1152917 | 1153270 | 1 | UrcA family protein |
|  | 1142309 | 1153869 | 1153555 | 1153869 | 1 | UrcA family protein |
| 7 | 1646533 | 1654985 | 1646533 | 1647243 | -1 | conserved protein of unknown function |
|  | 1646533 | 1654985 | 1647350 | 1648369 | 1 | protein of unknown function |
|  | 1646533 | 1654985 | 1648326 | 1648649 | -1 | Homeodomain-like domain-containing protein (fragment) |
|  | 1646533 | 1654985 | 1648555 | 1648779 | 1 | protein of unknown function |
|  | 1646533 | 1654985 | 1648837 | 1648929 | -1 | protein of unknown function |
|  | 1646533 | 1654985 | 1649179 | 1650015 | 1 | conserved protein of unknown function |
|  | 1646533 | 1654985 | 1650008 | 1650337 | 1 | conserved protein of unknown function |
|  | 1646533 | 1654985 | 1650337 | 1650849 | 1 | protein of unknown function |
|  | 1646533 | 1654985 | 1651133 | 1653322 | -1 | excinulease of nucleotide excision repair, DNA damage recognition component |
|  | 1646533 | 1654985 | 1653502 | 1653954 | 1 | CheY-like chemotaxis protein |
|  | 1646533 | 1654985 | 1653951 | 1654985 | -1 | Low specificity L-threonine aldolase |
| 8 | 1758103 | 1762173 | 1757519 | 1758106 | 1 | conserved protein of unknown function |
|  | 1758103 | 1762173 | 1758103 | 1758519 | 1 | conserved protein of unknown function |
|  | 1758103 | 1762173 | 1758516 | 1758932 | 1 | conserved protein of unknown function |
|  | 1758103 | 1762173 | 1758921 | 1759124 | -1 | conserved exported protein of unknown function |
|  | 1758103 | 1762173 | 1759182 | 1759469 | 1 | conserved protein of unknown function |
|  | 1758103 | 1762173 | 1759466 | 1759660 | 1 | Phage tail assembly chaperone |
|  | 1758103 | 1762173 | 1759657 | 1760202 | 1 | Phage tail tape measure protein |
|  | 1758103 | 1762173 | 1760199 | 1760816 | 1 | Glycoside hydrolase family 24 |
|  | 1758103 | 1762173 | 1760879 | 1761739 | 1 | Beta tubulin |
|  | 1758103 | 1762173 | 1761739 | 1762173 | 1 | Peptidase P60 |
| 9 | 2339938 | 2346051 | 2339938 | 2340078 | -1 | protein of unknown function |
|  | 2339938 | 2346051 | 2340518 | 2340982 | -1 | protein of unknown function |
|  | 2339938 | 2346051 | 2341270 | 2341374 | -1 | protein of unknown function |
|  | 2339938 | 2346051 | 2341663 | 2342670 | -1 | protein of unknown function |
|  | 2339938 | 2346051 | 2342667 | 2342915 | -1 | protein of unknown function |
|  | 2339938 | 2346051 | 2343138 | 2344070 | -1 | protein of unknown function |
|  | 2339938 | 2346051 | 2344207 | 2345334 | 1 | Site-specific integrase |
|  | 2339938 | 2346051 | 2345411 | 2345618 | 1 | protein of unknown function |
|  | 2339938 | 2346051 | 2345803 | 2346051 | 1 | protein of unknown function |
|  | 2339938 | 2344070 | 2339938 | 2340078 | -1 | protein of unknown function |
|  | 2339938 | 2344070 | 2340518 | 2340982 | -1 | protein of unknown function |
|  | 2339938 | 2344070 | 2341270 | 2341374 | -1 | protein of unknown function |
|  | 2339938 | 2344070 | 2341663 | 2342670 | -1 | protein of unknown function |
|  | 2339938 | 2344070 | 2342667 | 2342915 | -1 | protein of unknown function |
|  | 2339938 | 2344070 | 2343138 | 2344070 | -1 | protein of unknown function |
| 10 | 2669225 | 2682672 | 2669225 | 2669842 | -1 | 30S ribosomal protein S4 |
|  | 2669225 | 2682672 | 2670112 | 2670621 | 1 | conserved protein of unknown function |
|  | 2669225 | 2682672 | 2670657 | 2671013 | -1 | conserved protein of unknown function |
|  | 2669225 | 2682672 | 2671418 | 2674171 | 1 | Type II restriction enzyme, methylase subunit YeeA |
|  | 2669225 | 2682672 | 2674168 | 2675142 | 1 | conserved protein of unknown function |
|  | 2669225 | 2682672 | 2675129 | 2677183 | 1 | putative ATP-dependent helicase YeeB |
|  | 2669225 | 2682672 | 2677176 | 2678381 | 1 | conserved protein of unknown function |
|  | 2669225 | 2682672 | 2678407 | 2678769 | -1 | conserved protein of unknown function |
|  | 2669225 | 2682672 | 2678873 | 2679823 | 1 | transposase |
|  | 2669225 | 2682672 | 2680226 | 2680354 | 1 | protein of unknown function |
|  | 2669225 | 2682672 | 2680397 | 2680471 | -1 | protein of unknown function |
|  | 2669225 | 2682672 | 2680475 | 2681977 | -1 | conserved membrane protein of unknown function |
|  | 2669225 | 2682672 | 2681992 | 2682672 | -1 | conserved protein of unknown function |

**Supplementary Table 2:**

List of the identified genes involved in metal detoxification, hydrocarbon degradation and stress response.

| **Category** | **Subcategory** | **Subsystem** | **Role** |
| --- | --- | --- | --- |
| Virulence, Disease and Defense | Resistance to antibiotics and toxic compounds | Copper homeostasis | Multicopper oxidase |
| Virulence, Disease and Defense | Resistance to antibiotics and toxic compounds | Copper homeostasis | Cytochrome c heme lyase subunit CcmF |
| Virulence, Disease and Defense | Resistance to antibiotics and toxic compounds | Copper homeostasis | Cytochrome c heme lyase subunit CcmH |
| Virulence, Disease and Defense | Resistance to antibiotics and toxic compounds | Copper homeostasis | Copper-translocating P-type ATPase (EC 3.6.3.4) |
| Virulence, Disease and Defense | Resistance to antibiotics and toxic compounds | Copper homeostasis | Copper resistance protein B |
| Virulence, Disease and Defense | Resistance to antibiotics and toxic compounds | Cobalt-zinc-cadmium resistance | Heavy metal RND efflux outer membrane protein, CzcC family |
| Virulence, Disease and Defense | Resistance to antibiotics and toxic compounds | Cobalt-zinc-cadmium resistance | Zinc transporter ZitB |
| Virulence, Disease and Defense | Resistance to antibiotics and toxic compounds | Cobalt-zinc-cadmium resistance | Hypothetical protein involved in heavy metal export |
| Virulence, Disease and Defense | Resistance to antibiotics and toxic compounds | Cobalt-zinc-cadmium resistance | Probable Co/Zn/Cd efflux system membrane fusion protein |
| Virulence, Disease and Defense | Resistance to antibiotics and toxic compounds | Cobalt-zinc-cadmium resistance | Transcriptional regulator, MerR family |
| Virulence, Disease and Defense | Resistance to antibiotics and toxic compounds | Mercuric reductase | Mercuric ion reductase (EC 1.16.1.1) |
| Virulence, Disease and Defense | Resistance to antibiotics and toxic compounds | Mercury resistance operon | Mercuric ion reductase (EC 1.16.1.1) |
| Virulence, Disease and Defense | Resistance to antibiotics and toxic compounds | Copper homeostasis: copper tolerance | Copper homeostasis protein CutE |
| Virulence, Disease and Defense | Resistance to antibiotics and toxic compounds | Copper homeostasis: copper tolerance | Periplasmic divalent cation tolerance protein CutA |
| Virulence, Disease and Defense | Resistance to antibiotics and toxic compounds | Copper homeostasis: copper tolerance | Magnesium and cobalt efflux protein CorC |
| Virulence, Disease and Defense | Resistance to antibiotics and toxic compounds | Resistance to fluoroquinolones | DNA gyrase subunit B (EC 5.99.1.3) |
| Virulence, Disease and Defense | Resistance to antibiotics and toxic compounds | Resistance to fluoroquinolones | DNA gyrase subunit A (EC 5.99.1.3) |
| Virulence, Disease and Defense | Resistance to antibiotics and toxic compounds | Beta-lactamase | Beta-lactamase (EC 3.5.2.6) |
| Virulence, Disease and Defense | Resistance to antibiotics and toxic compounds | Beta-lactamase | Metal-dependent hydrolases of the beta-lactamase superfamily I |
| Virulence, Disease and Defense | Resistance to antibiotics and toxic compounds | Beta-lactamase | Probable beta-lactamase ybxI precursor (EC 3.5.2.6) |
| Virulence, Disease and Defense | Resistance to antibiotics and toxic compounds | Multidrug Resistance Efflux Pumps | RND efflux system, outer membrane lipoprotein, NodT family |
| Virulence, Disease and Defense | Resistance to antibiotics and toxic compounds | Multidrug Resistance Efflux Pumps | Multi antimicrobial extrusion protein (Na(+)/drug antiporter), MATE family of MDR efflux pumps |
| Virulence, Disease and Defense | Resistance to antibiotics and toxic compounds | Multidrug Resistance Efflux Pumps | Acriflavin resistance protein |
| Virulence, Disease and Defense | Resistance to antibiotics and toxic compounds | Multidrug Resistance Efflux Pumps | Multidrug efflux pump component MtrF |
| Virulence, Disease and Defense | Resistance to antibiotics and toxic compounds | Multidrug Resistance Efflux Pumps | Macrolide-specific efflux protein MacA |
| Virulence, Disease and Defense | Resistance to antibiotics and toxic compounds | Multidrug Resistance Efflux Pumps | Type I secretion outer membrane protein, TolC precursor |
| Virulence, Disease and Defense | Invasion and intracellular resistance | Mycobacterium virulence operon possibly involved in quinolinate biosynthesis | Quinolinate synthetase (EC 2.5.1.72) |
| Virulence, Disease and Defense | Invasion and intracellular resistance | Mycobacterium virulence operon possibly involved in quinolinate biosynthesis | Quinolinate phosphoribosyltransferase [decarboxylating] (EC 2.4.2.19) |
| Virulence, Disease and Defense | Invasion and intracellular resistance | Mycobacterium virulence operon possibly involved in quinolinate biosynthesis | L-aspartate oxidase (EC 1.4.3.16) |
| Virulence, Disease and Defense | Invasion and intracellular resistance | Mycobacterium virulence operon involved in protein synthesis (LSU ribosomal proteins) | LSU ribosomal protein L35p |
| Virulence, Disease and Defense | Invasion and intracellular resistance | Mycobacterium virulence operon involved in protein synthesis (LSU ribosomal proteins) | Translation initiation factor 3 |
| Virulence, Disease and Defense | Invasion and intracellular resistance | Mycobacterium virulence operon involved in protein synthesis (LSU ribosomal proteins) | LSU ribosomal protein L20p |
| Stress Response | Osmotic stress | Osmoregulation | Outer membrane protein A precursor |
| Stress Response | Oxidative stress | Protection from Reactive Oxygen Species | Superoxide dismutase [Cu-Zn] precursor (EC 1.15.1.1) |
| Stress Response | Oxidative stress | Protection from Reactive Oxygen Species | Superoxide dismutase [Fe] (EC 1.15.1.1) |
| Stress Response | Oxidative stress | Oxidative stress | Ferric uptake regulation protein FUR |
| Stress Response | Oxidative stress | Oxidative stress | Redox-sensitive transcriptional activator SoxR |
| Stress Response | Oxidative stress | Oxidative stress | Organic hydroperoxide resistance transcriptional regulator |
| Stress Response | Oxidative stress | Oxidative stress | Alkyl hydroperoxide reductase subunit C-like protein |
| Stress Response | Oxidative stress | Oxidative stress | Superoxide dismutase [Cu-Zn] precursor (EC 1.15.1.1) |
| Stress Response | Oxidative stress | Oxidative stress | Superoxide dismutase [Fe] (EC 1.15.1.1) |
| Stress Response | Oxidative stress | Oxidative stress | Phytochrome, two-component sensor histidine kinase (EC 2.7.3.-) |
| Stress Response | Oxidative stress | Oxidative stress | Organic hydroperoxide resistance protein |
| Stress Response | Oxidative stress | Glutathione: Biosynthesis and gamma-glutamyl cycle | Gamma-glutamyltranspeptidase (EC 2.3.2.2) |
| Stress Response | Oxidative stress | Glutathione: Biosynthesis and gamma-glutamyl cycle | Glutathione synthetase (EC 6.3.2.3) |
| Stress Response | Oxidative stress | Glutathione: Non-redox reactions | Glutathione S-transferase, zeta (EC 2.5.1.18) |
| Stress Response | Oxidative stress | Glutathione: Non-redox reactions | Glutathione S-transferase family protein |
| Stress Response | Oxidative stress | Glutathione: Non-redox reactions | Glutathione S-transferase (EC 2.5.1.18) |
| Stress Response | Oxidative stress | Glutathione: Non-redox reactions | Lactoylglutathione lyase (EC 4.4.1.5) |
| Stress Response | Oxidative stress | Glutathione: Non-redox reactions | Hydroxyacylglutathione hydrolase (EC 3.1.2.6) |
| Stress Response | Oxidative stress | Glutathione: Non-redox reactions | Glutathione S-transferase, unnamed subgroup (EC 2.5.1.18) |
| Stress Response | Oxidative stress | Glutathione: Non-redox reactions | Glutathione S-transferase, omega (EC 2.5.1.18) |
| Stress Response | Oxidative stress | Glutathione: Redox cycle | Glutathione reductase (EC 1.8.1.7) |
| Stress Response | Oxidative stress | Glutathione: Redox cycle | Uncharacterized monothiol glutaredoxin ycf64-like |
| Stress Response | Oxidative stress | Glutathione: Redox cycle | Glutaredoxin 3 (Grx2) |
| Stress Response | Oxidative stress | Glutaredoxins | Uncharacterized monothiol glutaredoxin ycf64-like |
| Stress Response | Oxidative stress | Glutaredoxins | Glutaredoxin 3 (Grx2) |
| Stress Response | Detoxification | Glutathione-dependent pathway of formaldehyde detoxification | S-formylglutathione hydrolase (EC 3.1.2.12) |
| Stress Response | Detoxification | Glutathione-dependent pathway of formaldehyde detoxification | S-(hydroxymethyl)glutathione dehydrogenase (EC 1.1.1.284) |
| Stress Response | Stress Response | Hfl operon | HflC protein |
| Stress Response | Stress Response | Hfl operon | RNA-binding protein Hfq |
| Stress Response | Stress Response | Hfl operon | HflK protein |
| Stress Response | Periplasmic Stress | Periplasmic Stress Response | Outer membrane protein H precursor |
| Stress Response | Periplasmic Stress | Periplasmic Stress Response | Intramembrane protease RasP/YluC, implicated in cell division based on FtsL cleavage |
| Stress Response | Periplasmic Stress | Periplasmic Stress Response | HtrA protease/chaperone protein |
| Cell Wall and Capsule | Capsular and extracellular polysacchrides | dTDP-rhamnose synthesis | dTDP-4-dehydrorhamnose reductase (EC 1.1.1.133) |
| Cell Wall and Capsule | Capsular and extracellular polysacchrides | dTDP-rhamnose synthesis | dTDP-glucose 4,6-dehydratase (EC 4.2.1.46) |
| Cell Wall and Capsule | Capsular and extracellular polysacchrides | dTDP-rhamnose synthesis | dTDP-4-dehydrorhamnose 3,5-epimerase (EC 5.1.3.13) |
| Cell Wall and Capsule | Capsular and extracellular polysacchrides | dTDP-rhamnose synthesis | Glucose-1-phosphate thymidylyltransferase (EC 2.7.7.24) |
| Cell Wall and Capsule | Capsular and extracellular polysacchrides | dTDP-rhamnose synthesis | dTDP-Rha:A-D-GlcNAc-diphosphoryl polyprenol, A-3-L-rhamnosyl transferase WbbL |
| Cell Wall and Capsule | Capsular and extracellular polysacchrides | Rhamnose containing glycans | UDP-glucose 4-epimerase (EC 5.1.3.2) |
| Cell Wall and Capsule | Capsular and extracellular polysacchrides | Rhamnose containing glycans | dTDP-4-dehydrorhamnose reductase (EC 1.1.1.133) |
| Cell Wall and Capsule | Capsular and extracellular polysacchrides | Rhamnose containing glycans | dTDP-glucose 4,6-dehydratase (EC 4.2.1.46) |
| Cell Wall and Capsule | Capsular and extracellular polysacchrides | Rhamnose containing glycans | dTDP-4-dehydrorhamnose 3,5-epimerase (EC 5.1.3.13) |
| Cell Wall and Capsule | Capsular and extracellular polysacchrides | Rhamnose containing glycans | Glucose-1-phosphate thymidylyltransferase (EC 2.7.7.24) |
| Metabolism of Aromatic Compounds | Peripheral pathways for catabolism of aromatic compounds | Quinate degradation | 3-dehydroquinate dehydratase II (EC 4.2.1.10) |
| Metabolism of Aromatic Compounds | Metabolism of central aromatic intermediates | Catechol branch of beta-ketoadipate pathway | Succinyl-CoA:3-ketoacid-coenzyme A transferase subunit A (EC 2.8.3.5) |
| Metabolism of Aromatic Compounds | Metabolism of central aromatic intermediates | Catechol branch of beta-ketoadipate pathway | Succinyl-CoA:3-ketoacid-coenzyme A transferase subunit B (EC 2.8.3.5) |
| Metabolism of Aromatic Compounds | Metabolism of central aromatic intermediates | Salicylate and gentisate catabolism | Fumarylacetoacetase (EC 3.7.1.2) |
| Metabolism of Aromatic Compounds | Metabolism of central aromatic intermediates | Salicylate and gentisate catabolism | Maleylacetoacetate isomerase (EC 5.2.1.2) |
| Metabolism of Aromatic Compounds | Metabolism of central aromatic intermediates | Salicylate and gentisate catabolism | Fumarylacetoacetate hydrolase family protein |
| Metabolism of Aromatic Compounds | Metabolism of central aromatic intermediates | Homogentisate pathway of aromatic compound degradation | Homogentisate 1,2-dioxygenase (EC 1.13.11.5) |
| Metabolism of Aromatic Compounds | Metabolism of central aromatic intermediates | Homogentisate pathway of aromatic compound degradation | Fumarylacetoacetase (EC 3.7.1.2) |
| Metabolism of Aromatic Compounds | Metabolism of central aromatic intermediates | Homogentisate pathway of aromatic compound degradation | 4-hydroxyphenylpyruvate dioxygenase (EC 1.13.11.27) |
| Metabolism of Aromatic Compounds | Metabolism of central aromatic intermediates | Homogentisate pathway of aromatic compound degradation | Maleylacetoacetate isomerase (EC 5.2.1.2) |
| Metabolism of Aromatic Compounds | Metabolism of Aromatic Compounds | Aromatic Amin Catabolism | Aldehyde dehydrogenase (EC 1.2.1.3), PaaZ |
| Metabolism of Aromatic Compounds | Metabolism of Aromatic Compounds | Aromatic Amin Catabolism | Nitrilotriacetate monooxygenase component B (EC 1.14.13.-) |
| Metabolism of Aromatic Compounds | Metabolism of Aromatic Compounds | Gentisate degradation | putative 4-hydroxybenzoyl-CoA thioesterase |
| Metabolism of Aromatic Compounds | Metabolism of Aromatic Compounds | Gentisate degradation | Maleylacetoacetate isomerase (EC 5.2.1.2) |
| Metabolism of Aromatic Compounds | Metabolism of Aromatic Compounds | Gentisate degradation | Fumarylacetoacetate hydrolase family protein |
| Metabolism of Aromatic Compounds | Metabolism of Aromatic Compounds | Chlorocyclohexane and chlorobenzene degradation | carboxymethylenebutenolidase [EC:3.1.1.45] |
| Metabolism of Aromatic Compounds | Metabolism of Aromatic Compounds | Chlorocyclohexane and chlorobenzene degradation | haloalkane dehalogenase [EC:3.8.1.5] |
| Metabolism of Aromatic Compounds | Metabolism of Aromatic Compounds | Benzoate degradation | p-hydroxybenzoate 3-monooxygenase [EC:1.14.13.2] |
| Metabolism of Aromatic Compounds | Metabolism of Aromatic Compounds | Benzoate degradation | 3-carboxy-cis,cis-muconate cycloisomerase [EC:5.5.1.2] |
| Metabolism of Aromatic Compounds | Metabolism of Aromatic Compounds | Benzoate degradation | 3-hydroxyacyl-CoA dehydrogenase EC:1.1.1.35 |
| Metabolism of Aromatic Compounds | Metabolism of Aromatic Compounds | Benzoate degradation | acetyl-CoA acyltransferase [EC:2.3.1.16] |
| Metabolism of Aromatic Compounds | Metabolism of Aromatic Compounds | Benzoate degradation | enoyl-CoA hydratase [EC:4.2.1.17] |
| Metabolism of Aromatic Compounds | Metabolism of Aromatic Compounds | Benzoate degradation | 4-hydroxy-4-methyl-2-oxoglutarate aldolase [EC:4.1.3.17] |
| Metabolism of Aromatic Compounds | Metabolism of Aromatic Compounds | Benzoate degradation | acetyl-CoA C-acetyltransferase [EC:2.3.1.9] |
| Metabolism of Aromatic Compounds | Metabolism of Aromatic Compounds | Fluorobenzoate degradation | carboxymethylenebutenolidase [EC:3.1.1.45] |
| Metabolism of Aromatic Compounds | Metabolism of Aromatic Compounds | Toluene degradation | 4-hydroxybenzaldehyde dehydrogenase (NADP+) [EC:1.2.1.96] |
| Metabolism of Aromatic Compounds | Metabolism of Aromatic Compounds | Toluene degradation | 2-[hydroxy(phenyl)methyl]-succinyl-CoA dehydrogenase BbsC subunit [EC:1.1.1.35] |
| Metabolism of Aromatic Compounds | Metabolism of Aromatic Compounds | Chloroalkane and chloroalkene degradation | alcohol dehydrogenase, propanol-preferring [EC:1.1.1.1] |
| Metabolism of Aromatic Compounds | Metabolism of Aromatic Compounds | Chloroalkane and chloroalkene degradation | soluble epoxide hydrolase EC:3.3.2.10 |
| Metabolism of Aromatic Compounds | Metabolism of Aromatic Compounds | Naphthalene degradation | 2-hydroxychromene-2-carboxylate isomerase [EC:5.99.1.4] |
| Metabolism of Aromatic Compounds | Metabolism of Aromatic Compounds | Naphthalene degradation | salicylate hydroxylase [EC:1.14.13.1] |
| Metabolism of Aromatic Compounds | Metabolism of Aromatic Compounds | Aminobenzoate degradation | amidase [EC:3.5.1.4] |
| Metabolism of Aromatic Compounds | Metabolism of Aromatic Compounds | Aminobenzoate degradation | aryldialkylphosphatase EC 3.1.8.1 |
| Metabolism of Aromatic Compounds | Metabolism of Aromatic Compounds | Aminobenzoate degradation | acetoacetate CoA-transferase alpha subunit EC. 2.8.3.9 |
| Metabolism of Aromatic Compounds | Metabolism of Aromatic Compounds | Aminobenzoate degradation | enoyl-CoA hydratase [EC:4.2.1.17] |
| Metabolism of Aromatic Compounds | Metabolism of Aromatic Compounds | Ethylbenzene degradation | acetyl-CoA acyltransferase [EC:2.3.1.16] |
